# New Furoisocoumarins with Phytotoxic Activity from the Fungus *Aspergillus calidoustus* VKM F-4916

**DOI:** 10.3390/toxins18050234

**Published:** 2026-05-20

**Authors:** Tatiana V. Antipova, Vsevolod R. Dubovik, Anton N. Yurchenko, Olesya I. Zhuravleva, Valentina P. Zhelifonova, Elizaveta G. Lukina, Boris P. Baskunov, Oussama Abdelhamid Mammeri, Sergey N. Smirnov, Natalya E. Ivanushkina, Kirill V. Zaitsev, Qunfang Weng, Mikhail B. Vainshtein, Alexander O. Berestetskiy

**Affiliations:** 1 G. K. Skryabin Institute of Biochemistry and Physiology of Microorganisms, Federal Research Center Pushchino Scientific Center for Biological Research of the Russian Academy of Sciences, Pushchino 142290, Russia; zhelifonova@yandex.ru (V.P.Z.); baskunov@pbcras.ru (B.P.B.); ivanushkina58@mail.ru (N.E.I.); vain@pbcras.ru (M.B.V.); 2 All-Russian Institute of Plant Protection, Saint-Petersburg 196608, Russia; vdubovik@vizr.spb.ru (V.R.D.); yurchenkoan@piboc.dvo.ru (A.N.Y.); elizaveta121999@mail.ru (E.G.L.); aberestetskiy@vizr.spb.ru (A.O.B.); 3 G.B. Elyakov Pacific Institute of Bioorganic Chemistry, Far Eastern Branch of the Russian Academy of Sciences, Vladivostok 690022, Russia; zhuravleva_oi@piboc.dvo.ru; 4 Institute of Chemistry, Saint-Petersburg State University, Saint-Petersburg 199034, Russia; oussama.mammeri@mail.ru (O.A.M.); sergey.smirnov@spbu.ru (S.N.S.); 5 Department of Chemistry, Lomonosov Moscow State University, Moscow 119991, Russia; zaitsev@org.chem.msu.ru; 6 Faculty of Plant Protection, South China Agricultural University, Guangzhou 510642, China; wengweng@scau.edu.cn

**Keywords:** isocoumarins, asperisocoumarin J, phytotoxicity, Pesti-DGI-Net online resource

## Abstract

*Aspergillus* fungi are a source of low-molecular compounds of various structures possessing biological activities. We investigated the secondary metabolite profile of the soil fungus *A. calidoustus* VKM F-4916. The strain was found to synthesize new metabolites attributed to furoisocoumarins, which we named asperisocoumarin J and K, and a known siderophore desferritriacetylfusigen. The structure of asperisocoumarin J and K were determined by mass spectrometry and NMR spectroscopy. Asperisocoumarins J, K and desferritriacetylfusigen possessed a phytotoxicity, inhibiting the lettuce root growth. Sow thistle leaf and wheat leaf cuttings were sensitive to the action of asperisocoumarin J and K at a concentration of 5 mg/mL. Analysis of the structures of furoisocoumarins (asperisocoumarins J and K) using the online resource Pesti-DGI-Net showed that compounds had the physico-chemical properties favorable for pesticide development, in particular, fungicides and herbicides. An in-depth study of the phytotoxic properties of furoisocoumarins and their natural analogs is of interest in the context of the search for new herbicide compounds.

## 1. Introduction

Fungi of the genus *Aspergillus* are well known for the production of numerous and diverse secondary metabolites with toxic or, in contrast, therapeutic potential [1]. Some of these metabolites can also be used as chemotaxonomic indicators to identify fungal species [2,3]. Structurally, these compounds belong to terpenoids, alkaloids, polyketides, etc. Several types of polyketides have been found in *Aspergillus* fungi, among which isocoumarins with various structural features are important natural compounds [4]. Although the structures of many secondary metabolites have already been established, chemotaxonomic studies have discovered many *Aspergillus* species that contain uncharacterized or unknown metabolites. Research on the latter is a way to discover new biologically active compounds of interest for various areas of the economy [5]. Recently, a promising area of research in plant protection against harmful organisms has been the search for natural compounds with pesticidal properties, since the excessive dependence of plant production on synthetic agrochemicals has led to negative consequences for humans and the environment [6,7].

The *Aspergillus* section *Usti* presents a huge diversity of representatives in various soils; they are also present in the air, on food products, plant residues, and industrial materials, and are known as causative agents of invasive infections in people with weakened immune systems [8,9]. Until the last decade, identification of *A. ustus* was based on phenotypic characteristics only, while high morphological variability was also observed between different strains of the same species. In recent years, the *Aspergillus* section *Usti* has been investigated with molecular tools, which have enabled the identification of new species within it [2]. These studies, along with molecular markers, used some species-specific markers, such as growth on selective media at a specific temperature, and features of the secondary metabolite spectrum. As a result, the *A. ustus* sensu lato species was divided into several, including *A. ustus* and *A. calidoustus*, with only the latter growing at 37 °C. During recent decades, studies have shown that clinical isolates of *A. calidoustus* have been obtained from immunocompromised patients, mainly during lung transplantation with azole treatment [10]. Austins, drimanes, ophiobolins G and H, TMC-120 B, and three unidentified compounds are considered to be chemotaxonomic markers among the secondary metabolites of *A. calidoustus* [2]. These metabolites have shown a wide diversity of biological activities. Further studies on *A. calidoustus* strains are, therefore, of significant interest to the research community.

To search for pesticide molecules, computational methods of chemo- and bioinformatics, tested for creating new drugs, are actively used. Several predictors that allow the selection of promising molecules have already been proposed, using existing experience in the creation of chemical pesticides and taking into account their biological activities, physicochemical properties, and mobility in plants [11,12]. To quantitatively assess pesticide likeness, a scoring function that includes a quantitative assessment of insecticide, fungicide, and herbicide likeness was established [13]. To date, an online platform, CoPLE, based on Pesti-DGI-Net has been developed. Pesti-DGI-Net serves as a valuable tool for the rational design of pesticide molecules [11].

This study is dedicated to the biosynthesis of secondary metabolites by *A. calidoustus* VKM F-4916 and their biological activity. The structures of new metabolites belonging to furoisocoumarins were established using modern physicochemical methods.

## 2. Results

### 2.1. Characteristics of the Strain

The cultural, morphological (phenotypic), and physiological characteristics of the VKM F-4916 strain revealed full compliance with the diagnostic features of the species *A. calidoustus*, including good growth at 37 °C (CYA 25–26 mm) and the violet coloration of agar blocks with mycelium using Ehrlich’s reagent [14]. At 25 °C, the diameters of colonies on the 7th day of growth were as follows: CYA, 30–31 mm; MEA, 44–45 mm; and YES, 35–36 mm. On CYA at 25 °C, the colonies were dense, woolly or velvety, and brown, and the reverse side was dull yellow. Conidiation was abundant; heads were spherical, loose, and disheveled; vesicles were 7–11 μm, spherical to elongated; and conidia were spherical, coarsely textured, and rough without spines, measuring 2.6–3.5 μm in diameter. The results of genotyping were consistent with the phenotypic study data of the culture. A high level of similarity of all sequences with the *A. calidoustus* type strain CBS 121601 (*CaM*, 100%; *BenA*, 99.77%; *RPB2*, 100%) and the reference strain *A. calidoustus* CBS 113228 (ITS, 99.89%) was demonstrated. All these data allowed us to identify VKM F-4916 as an *A. calidoustus* strain.

### 2.2. Determination of Secondary Metabolites from A. calidoustus Extract

**Figure 1:**
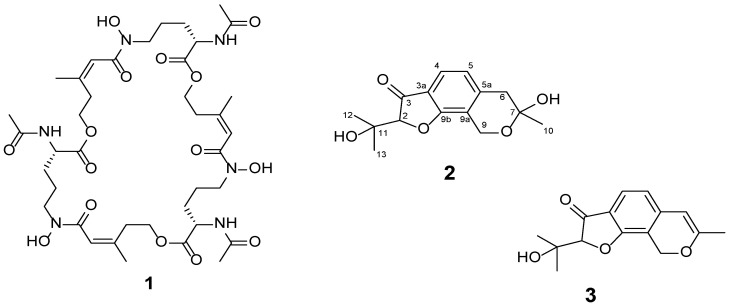
Structures of isolated compounds.

The extract contained the following metabolites (Table 1). Metabolite **1** reacted with the FeCl_3_ reagent to yield a brown coloration. No characteristic absorption bands in the UV spectrum were observed. Detection of molecular ions at 853 [M + H]^+^ and 851 [M − H]^−^, along with the sequential loss of *m*/*z* 284 fragments in the MS/MS spectra, indicated that this metabolite is formed by condensation of three identical-mass subunits. Based on its characteristics, metabolite **1** was identified as desferritriacetylfusigen (Figure 1). For metabolite **2**, absorption bands at λ_max_ 217, 265, and 329 nm were present in the UV spectrum; in the mass spectrum, molecular ions at 279 [M + H]^+^ and 277 [M − H]^−^ were observed. Metabolite **3** had absorption bands at λ_max_ 214, 291, and 331 nm and a molecular mass of 260 Da.

### 2.3. Structure Elucidation of New Furoisocoumarins

Metabolite **2** was isolated as a yellowish amorphous powder. The molecular formula of the metabolite was determined as C_15_H_18_O_5_ based on a HR(+)ESI MS peak [M + Na]^+^ at *m*/*z* 301.1044 (calculated for C_15_H_18_O_5_Na 301.1046) and confirmed by ^13^C NMR spectral data; this formula corresponds to seven degrees of unsaturation.

A preliminary study of the ^1^H and ^13^C NMR spectra of compound **2** showed values close to those of asperisocoumarins [15]. According to the ^1^H NMR spectrum (Table 2, Figure S1), the molecule contained two aromatic protons H-4 and H-5 (δ_H_ 7.47 and 6.84 ppm) appearing as doublets (*J* = 8.0 Hz), two aliphatic methylene groups H_2_C-6 (δ_H_ 2.93, 3.00 ppm) and H_2_C-9 (δ_H_ 4.96, 4.88 ppm), one oxymethine proton H-2 (δ_H_ 4.37 ppm) and three methyl groups Me-10, Me-12 and Me-13 appearing as singlets (δ_H_ 1.60, 1.35, and 1.21 ppm).

According to ^13^C NMR spectral data (Table 2, Figure S2), the molecule contains 15 carbon atoms: one carbonyl carbon atom C-3 (δ_C_ 200.6 ppm), six aromatic carbon atoms C-3a, C-4, C-5, C-5a, C-9a, and C-9b (δ_C_ 119.55, 121.97, 123.40, 143.24, 119.30, and 169.1 ppm, respectively), two methylene carbon atoms C-6 and C-9 (δ_C_ 38.80 and 57.6 ppm), two quaternary carbon atoms C-7 and C-11 (δ_C_ 94.84 and 72.57 ppm), one oxymethine carbon atom C-2 (δ_C_ 89.82 ppm) and three methyl carbon atoms C-10, C-12 and C-13 (δ_C_ 29.4, 25.93, and 24.4 ppm).

**Figure 2:**
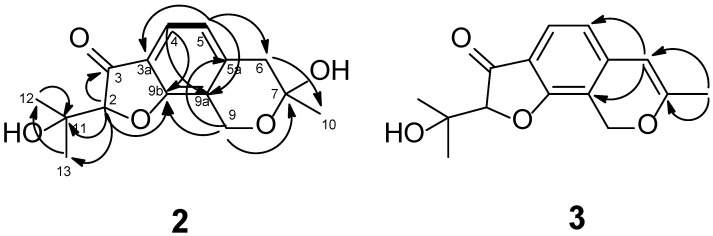
The key HMBC (arrows) and ^1^H–^1^H COSY correlations in the compounds **2** and **3**.

The HMBC correlations (Figure 2 and Figure S5) from H-2 to C-3 and C-9b, from H-4 to C-3, C-9b, and C-5a, from H-5 to C-3a, C-9a, and C-6, from H_2_-9 to C-5a, C-7, C-9a, and C-9b, and from H_2_-6 to C-5a, C-9a, and C-7 together with coupling constant ^3^J = 7.9 Hz between H-4 and H-5 confirmed the furoisocoumarin skeleton. HMBC correlations from H_3_-10 to C-7 and C-6, from H-2 to C-11, C-12, and C-13, from H_3_-12 to C-2, C-11, and C-12, and H_3_-13 to C-2, C-11, and C-13 indicated the location of Me-10 at C-7, and hydroxyisopropyl side chain at C-2. The presence of OH groups at C-7 and C-11 was suggested based on characteristic downfield shifts of these carbons. Thus, the structure of compound **2** was established as 7-hydroxy-2-(2-hydroxypropan-2-yl)-7-methyl-6,9-dihydro-7H-furo[3,2-*h*]isochromen-3(2H)-one, and it has been named asperisocoumarin J.

It should be noted that most of the signals in the ^13^C NMR spectrum are binary (Table 2, Figure S2). A similar split is also observed for H-2 and one of the H-9 signals (Table 2, Figure S1). This suggests that compound **2** is a mixture of two stereoisomers in a ratio of 1:1. Unfortunately, attempts to separate these stereoisomers using HPLC were unsuccessful.

The molecular formula of compound **3** was determined as C_15_H_16_O_4_ based on the HR(+)ESI MS containing the peak of the protonated molecule [M + H]+ (*m*/*z* 261.1120), which was confirmed by ^13^C NMR spectral data. The ^1^H and ^13^C NMR spectra of compound **3** (Table 2, Figures S6–S10) were very similar to those for compound **2**, with the exception of the proton and carbon signals at C-5, C-5a, C-6, C-7, C-9, C-9a, and C-10 of the isochromene ring. The presence of an *sp^2^* methine and a quaternary *sp^2^* carbon in the NMR spectra of compound **3** instead of an *sp^3^* methylene and an oxygenated quaternary *sp^3^* carbon in compound **2**, together with MS data and HMBC correlations (Figure 2 and Figure S11) from H_3_-10 (δ_H_ 1.98) to C-7 (δ_C_ 160.7) and C-6 (δ_C_ 101.7), and from H-6 (δ_H_ 5.69) to C-5 (δ_C_ 117.6), C-5a (δ_C_ 109.0), C-7, and C-10 (δ_C_ 20.1) revealed the structure of compound **3** as 2-(2-hydroxypropan-2-yl)-7-methyl-6,9-dihydro-7H-furo[3,2-*h*]isochromen-3(2H)-one, and it has been named asperisocoumarin K.

Unfortunately, compound 3 turned out to be unstable and completely degraded after the NMR spectra were obtained. For this reason, it was not possible to obtain the CD spectrum of this compound for further determination of the stereostructure.

### 2.4. Biological Activity of Secondary Metabolites

The antimicrobial activity study showed that these metabolites had no activity against *Bacillus subtilis*, *Escherichia coli*, or *Candida albicans*.

In the growth inhibition assay, asperisocoumarin J, asperisocoumarin K, and desferritriacetylfusigen at a concentration of 500 μg/mL suppressed the root growth of lettuce seedlings by 92%, 85%, and 87%, respectively.

In the leaf disc/segment-puncture assay, perennial sowthistle and wheat were sensitive to the action of asperisocoumarin J and asperisocoumarin K at a concentration of 5 mg/mL: the necrosis diameter in sowthistle leaf discs was 4.3 and 5.3 mm, and in wheat leaf segments, 1.1 and 3.3 mm (Figure S14), respectively. At a concentration of 1 mg/mL, the compounds demonstrated no phytotoxicity. Desferritriacetylfusigen was not phytotoxic in this bioassay.

### 2.5. Assessment of the Pesticide-likeness of Metabolites to Pesticides Using an Online Resource Pesti-DGI-Net

In this section, the structures of furoisocoumarin-type metabolites—asperisocoumarin J and K—were analyzed using the Pesti-DGI-Net online resource (Table 3). It was shown that asperisocoumarin J and K had the properties of pesticides, in particular, of fungicides and herbicides. Asperisocoumarin K (**3**) had a higher pesticide likeness coefficient (0.899) as compared to asperisocoumarin J (**2**) (0.765) and approximately the same level of herbicide likeness (0.818–0.820). The pesticidal activity of the detected metabolites is apparently due to the presence of the isochroman core, which has a high pesticide likeness coefficient (0.998) (Table 3).

## 3. Discussion

It is known that coumarins, isocoumarins, and their derivatives have attracted considerable interest in the field of developing new biorational herbicides due to their biological activities [16,17,18,19]. Phytotoxic and alleloherbicide activities have also been demonstrated for furanocoumarins [17]. The new metabolites, asperisocoumarin J and K, were attributed to furoisocoumarins. Furoisocoumarins are secondary metabolites that are relatively rare in fungi [20]. Most often, these metabolites are found in the genera *Penicillium* and *Aspergillus* [15,21,22,23,24,25]. These compounds have been shown to exhibit a variety of biological activities, including antibacterial, phytotoxic, and cytotoxic effects. Some asperisocoumarins have been shown to have moderate α-glucosidase inhibitory activity and antibacterial activity against *Salmonella* [15,24]. Pergillin has been shown to have approximately 50% inhibitory activity on wheat coleoptiles at a concentration of 10^−3^ M [21]. The addition of a double bond at C12–C13 to form dihydropergillin increases the inhibitory activity on wheat coleoptiles to 100% at a similar concentration [26]. Pseudodeflectusin, which differs from pergillin in the position of the hydroxyl group on the isochroman ring, exhibits cytotoxicity against several gastric, cervical, and peripheral blood cancer cell lines at 39 µM [23].

Desferritriacetylfusarin (desferritriacetylfusigen) is a cyclic triester belonging to the siderophore family. It consists of three *N^5^*-*cis*-anhydromevalonyl-*N^5^*-hydroxy-*N^2^*-acetyl-*L*-ornithine units connected via ester linkages and readily binds iron ions to yield triacetylfusarinine C (triacetylfusigen) [27,28]. Previously, four strains of *A. calidoustus* were found to synthesize it; apparently, this metabolite forms due to a lack of iron ions in the culture medium [9]. Desferritriacetylfusarin has previously been shown to exhibit antibiotic activity against *Brevibacillus brevis*, *Pseudomonas fluorescens*, *Micrococcus luteus*, and *Proteus species* [27]. Triacetylfusarinine C, a key metabolite produced by *Ilyonectria* fungi, serves as a critical link to root rot disease virulence in American ginseng (*Panax quinquefolius*) [29].

The study showed that *A. calidoustus* strain VKM F-4916 is a producer of asperisocoumarin J, K, and desferritriacetylfusarin, which exhibit phytotoxic activity. Pesticidal properties of these metabolites are theoretically predicted. Therefore, *A. calidoustus* strain VKM F-4916 forms secondary metabolites that can be considered potential biopesticides (herbicides) and their combined use may also enhance these properties through a synergistic effect.

## 4. Conclusions

Thus, the strain *A. calidoustus* VKM F-4916 synthesizes furoisocoumarins—the new metabolites asperisocoumarin J (**2**) and K (**3**), as well as the siderophore desferritriacetylfusigen (**1**). Analysis of the structures of asperisocoumarins J and K using the Pesti-DGI-Net online resource showed that all compounds had pesticidal properties, in particular fungicidal and herbicidal ones, which was due to the presence of the isochroman core. Our experiments showed asperisocoumarin J, K, and desferritriacetylfusigen to have phytotoxic activity. An in-depth assessment of the herbicidal potential of these furoisocoumarins and their analogs would be of interest in the future.

## 5. Materials and Methods

### 5.1. Strain

The object of the study was the *A. calidoustus* strain VKM F-4916, isolated from soil in the Krasnodar Territory, Russia, and stored in the All-Russian Collection of Microorganisms (VKM). Phenotypic characteristics were determined during cultivation on nutrient media: Czapek yeast extract agar (**CYA**), malt extract agar (**MEA**), yeast extract sucrose agar (**YES**) at a temperature of 25 °C, and on CYA at 37 °C for 7 days. Macroscopic (colony morphology, color, diameter) and microscopic (reproductive structures, shape and size of conidia) features were assessed [14]. Genotypic characteristics were determined using the universal fungal barcode of the internal transcribed spacer region (**ITS**), as well as three housekeeping genes encoding calmodulin (**CaM**), β-tubulin (**BenA**), and RNA polymerase II (**RPB2**) [30]. The obtained sequences were deposited in GenBank (ITS, PV799291; RPB2, PV839324; BenA, PV839325; CaM, PV839326).

### 5.2. Cultivation of the Strain

The strain was grown in a medium of the following composition (g/L distilled water): mannitol, 50.0; succinic acid, 5.4; MgSO_4_·7H_2_O, 0.3; KH_2_PO_4_, 1.0; ZnSO_4_·7H_2_O, 0.0066; pH was adjusted to 5.4 with a 25% NH_4_OH solution. The fungus was grown submerged on a shaker (220 rpm) at 24 ± 1 °C in 750 mL flasks containing 150 mL of liquid medium. The medium was inoculated with an aqueous spore suspension (1–2 × 10^6^ spores/mL) obtained from 14-day cultures grown on the surface of slanted malt extract agar. Cultivation was continued for 12 days.

### 5.3. Isolation and Identification of the Secondary Metabolites

The metabolites of the strain were extracted from the culture liquid filtrate (1.075 L) by three-fold extraction with chloroform. The chloroform extract was dried over anhydrous Na_2_SO_4_, filtered, and evaporated to dryness on a rotary evaporator under reduced pressure. The resulting precipitate was analyzed by physicochemical methods.

The extract was analyzed by TLC on silica gel plates (Silica gel F254, Merck, Darmstadt, Germany) using the solvent system CHCl_3_/MeOH (9:1). Metabolites were detected by absorption and fluorescence at 254 and 366 nm and after spraying the plates with 5% FeCl_3_ in methanol. The extracts were also analyzed by HPLC-MS-UV using an Acquity UPLC H-class chromatograph (Waters Corp., Milford, MA, USA) equipped with a diode array detector and a single quadrupole mass spectrometric detector (QDa, Waters Corp., Milford, MA, USA). A UPLC Acquity BEH C18 column (Waters Corp., Milford, MA, USA) measuring 2.1 × 50 mm with a particle size of 1.7 μm was used to separate the extracts. The dry residue of the extracts (1 mg) was dissolved in 1000 μL of acetonitrile. The extracts were eluted with a mixture of acetonitrile and 0.1% formic acid using a linear acetonitrile gradient (from 10 to 100% acetonitrile over 5 min). The eluent flow rate was 500 μL/min; the column temperature was 40 °C. Substances were detected in the wavelength range of 190–600 nm and over the *m*/*z* range of 100–1000 Da in positive and negative ion modes. MS spectra of the compounds were recorded using an LCQ Advantage MAX quadrupole mass spectrometer (Thermo Finnigan, Bremen, Germany) equipped with a single-channel syringe pump for direct sample injection into the atmospheric pressure chemical ionization (APCI) chamber. Preparative HPLC was performed on an Agilent 1100 chromatograph (Agilent Technologies, Santa Clara, CA, USA) equipped with an Agilent 1100 refractometer (Agilent Technologies, Santa Clara, CA, USA) using a YMC-Pack Pro C18 column (250 mm × 4.6 mm, YMC Co., Ishikawa, Japan). High-resolution mass spectra were obtained using a Shimadzu Nexera X2 LCMS-9030 quadrupole time-of-flight mass spectrometer (Shimadzu Corp., Kyoto, Japan) and a MaXis impact mass spectrometer (Bruker Daltonics GmbH, Rheinstetten, Germany).

The extract (243 mg) was loaded onto a column (3 × 7 cm) packed with silica gel (Silica gel 60, 0.063–0.1 mm, Merck, Germany). Elution was performed with a gradient of CHCl_3_/MeOH (100:0 to 1:1, *v*/*v*) to obtain six fractions (1–6). Fraction 2 was further separated by Sephadex LH-20 chromatography (CHCl_3_/MeOH, 1:1), which led to the purification of compound 2 (97 mg). Fraction 3 was purified by preparative TLC (plate: 20 × 20 cm; developing solvent: chloroform–methanol, 9:1) and then by HPLC (CH_3_CN–H_2_O, 40:60) to yield compound 3 (4.5 mg). Fraction 6 contained compound 1 (95 mg).

### 5.4. Establishment of the Structure of a New Furoisocoumarin

UV spectra of the compounds in methanol were obtained on a UV-160A spectrophotometer (Shimadzu Corp., Kyoto, Japan). NMR spectra were recorded in CDCl_3_ on a Bruker AVANCE III 400 MHz spectrometer (Bruker BioSpin GmbH, Rheinstetten, Germany) and a Bruker Avance III-500 spectrometer (Bruker BioSpin GmbH, Rheinstetten, Germany). The residual solvent signal (δ_H_ 7.26 ppm) for ^1^H NMR spectra and the CDCl_3_ carbon signal (δ_C_ 77.16 ppm) for ^13^C NMR spectra were used as reference values. COSY, HSQC, and HMBC experiments were performed using standard Bruker pulse programs. High-resolution mass spectra were obtained using a Shimadzu Nexera X2 LCMS-9030 quadrupole time-of-flight mass spectrometer (Japan) and a MaXis impact mass spectrometer (Bruker Daltonics GmbH, Rheinstetten, Germany).

**Asperisocoumarin J (2):** UV (MeOH) λ_max_ (log ε) 215 (4.04), 265 (3.75), 329 (3.42) nm; ^1^H and ^13^C NMR data, see Figures S1–S5; HRESIMS *m*/*z* 301.1044 [M + Na]^+^ (calcd. for C_15_H_18_O_5_Na 301.1046, Δ −0.8 ppm) (Figure S12, Supplementary Materials).

**Asperisocoumarin K (3):** UV (MeOH) λ_max_ (log ε) 214 (4.16), 291 (3.81), 331 (3.06) nm; ^1^H and ^13^C NMR data, see Figures S6–S11; HRESIMS *m*/*z* 261.1120 [M + H]^+^ (calcd. for C_15_H_17_O_4_ 261.1121, Δ −0.5 ppm) (Figure S13, Supplementary Materials).

### 5.5. Evaluation of Biological Activity

The antimicrobial activity of desferritriacetylfusigen (**1**), asperisocoumarin J (**2**), and asperisocoumarin K (**3**) at a concentration of 500 μg per disk was studied against three microorganisms: *Bacillus subtilis* (ATCC 6633), *Escherichia coli* (ATCC 25922), and the yeast *Candida albicans* (NCPF 3179) using the disk diffusion method [31,32].

The phytotoxic activity of the isolated compounds was evaluated by growth inhibitory assay using lettuce (*Lactuca sativa*) seeds at a concentration of 500 μg/mL, and by leaf-puncture assay using leaf discs of perennial sowthistle (*Sonchus arvensis*) and leaf segments of common wheat (*Triticum aestivum*) at concentration of 1 and 5 mg/mL as previously described [31,32].

Pesticide likeness of the metabolites was assessed using the online resource Pesti-DGI-Net [11].

### 5.6. Statistical Analysis

The data obtained were subjected to various statistical procedures, including calculation of mean and standard error, using Excel for Windows 16.0.

## Figures and Tables

**Table 1:** Physico-chemical properties of *A. calidoustus* secondary metabolites.

Entry	Metabolites	UV Spectrum, *λ*_max,_ nm	Collision Energy/Molecular Ions and Characteristic Peaks in MS/MS Spectra (% of Intensity)	
			[M − H]^−^	[M + H]^+^
**1**	Desferritriacetylfusigen	210	25/851 (72), 739 (6), 679 (7), 568 (100), 457 (8), 283 (11)	22/853 (100), 835 (41), 741 (97), 681 (5), 569 (41), 457 (4)
**2**		215, 265, 329	19/277 (90), 219 (100)	18/279 (70), 261 (25), 206 (100)
**3**		214, 291, 331	27/259 (100), 241 (30), 231 (70), 217 (88), 199 (40)	18/261 (60), 243 (82), 203 (100)

**Table 2:** ^1^H and ^13^C NMR spectral data of asperisocoumarin J (**2**) and asperisocoumarin K (**3**) in CDCl_3_.

No	2 ^a^		3 ^b^	
	δ_C_, ppm	δ_H_, ppm (*J* in Hz)	δ_C_, ppm	δ_H_, ppm (*J* in Hz)
2	89.82/89.76, CH	4.37, s/4.36, s	89.7, CH	4.37, s
3	200.6, C		199.9, C	
3a	119.55/119.54, C		119.7, C	
4	121.97/121.95, CH	7.46, d (7.9)	124.3, CH	7.47, d (7.9)
5	123.40/123.39, CH	6.82, d (7.9)	117.6, CH	6.63, d (7.9)
5a	143.24/143.16, C		109.0, C	
6	38.80/38.79, CH_2_	a: 2.93, d (17.4)	101.7, CH	5.69, s
		b: 3.00, d (17.4)		
7	94.84/94.81, C		160.7, C	
9	57.6, CH_2_	4.96, d (16.0) 4.88, d (15.6)/4.87, d (16.0)	62.8, CH_2_	5.22, d (13.2) 5.28, d (13.2)
9a	119.30/119.29, C		142.6, C	
9b	169.1, C		168.0, C	
10	29.4/29.3, CH_3_	1.60, s	20.1, CH_3_	1.98, s
11	72.57/72.56, C		72.6, C	
12	25.93/25.88, CH_3_	1.35, br s	26.2, CH_3_	1.37, s
13	24.4/24.2, CH_3_	1.21, s	24.1, CH_3_	1.19, s

^a^ The ^1^H and ^13^C NMR spectra were obtained at 400 MHz and 100 MHz, respectively. ^b^ The ^1^H and ^13^C NMR spectra were obtained at 500 MHz and 125 MHz, respectively.

**Table 3:** Analysis of the relationship between metabolite structures and pesticidal properties using the online resource Pesti-DGI-Net.

Metabolite	Double Interpretability *		Likeness Probability Forecast Coefficient			
	Graph Influence Mechanism	Grad-CAM	Pesticide	Fungicide	Insecticide	Herbicide
Asperisocoumarin J (**2**)	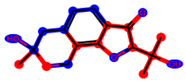	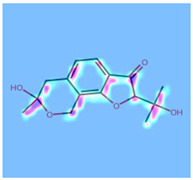	0.765	0.620	0.389	0.818
Asperisocoumarin K (**3**)	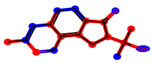	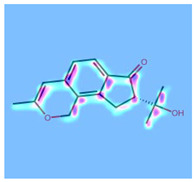	0.899	0.591	0.357	0.820
Isochroman	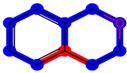	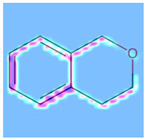	0.998	0.862	0.624	0.995

* Pesti-DGI-Net used the graph influence mechanism and Grad-CAM (gradient-weighted class activation mapping) to visualize the prediction results, where red regions in molecular graphs and highlighted regions in molecular images were recognized as key substructures.

## Data Availability

The original contributions presented in this study are included in the article/Supplementary Material. Further inquiries can be directed to the corresponding author.

## Supplementary Materials

The following supporting information can be downloaded at: https://www.mdpi.com/article/10.3390/toxins18050234/s1, Figure S1: ^1^H NMR spectrum (400 MHz, CDCl_3_) of asperisocoumarin J (**2**); Figure S2: ^13^C NMR spectrum (100 MHz, CDCl_3_) of asperisocoumarin J (**2**); Figure S3: ^1^H−^1^H COSY NMR spectrum (400 MHz, CDCl_3_) of asperisocoumarin J (**2**); Figure S4: HSQC NMR spectrum (400 MHz, CDCl_3_) of asperisocoumarin J (**2**); Figure S5: HMBC NMR spectrum (400 MHz, CDCl_3_) of asperisocoumarin J (**2**); Figure S6: ^1^H NMR spectrum (500 MHz, CDCl_3_) of asperisocoumarin K (**3**); Figure S7: ^13^C NMR spectrum (125 MHz, CDCl_3_) of asperisocoumarin K (**3**); Figure S8: DEPT-135 spectrum (125 MHz, CDCl_3_) of asperisocoumarin K (**3**); Figure S9: ^1^H−^1^H COSY NMR spectrum (500 MHz, CDCl_3_) of asperisocoumarin K (**3**); Figure S10: HSQC NMR spectrum (500 MHz, CDCl_3_) of asperisocoumarin K (**3**); Figure S11: HMBC NMR spectrum (500 MHz, CDCl_3_) of asperisocoumarin K (**3**); Figure S12: HR (+) ESI mass spectrum of asperisocoumarin J (2); Figure S13: HR (+) ESI mass spectrum of asperisocoumarin K (**3**); Figure S14. The necrosis of sowthistle leaf discs and wheat leaf segments under action asperisocoumarins J (**2**) (a) and K (**3**) (b).
