# Supplementary material for: New Furoisocoumarins with Phytotoxic Activity from the Fungus Aspergillus calidoustus VKM F-4916
Source: Toxins (Basel). 2026 May 20;18(5):234. doi: 10.3390/toxins18050234 (PMC13211676; doi:10.3390/toxins18050234)
Supplement: Supplementary file 1 [file toxins-18-00234-s001.zip › toxins-4301355-supplementary.pdf]

Supplementary Materials:

Table of Contents

Figure S1. ^{1}H NMR spectrum (400 MHz, $CDCl_3$) of asperisocoumarin J (**2**)....3

Figure S2. ^{13}C NMR spectrum (100 MHz, $CDCl_3$) of asperisocoumarin J (**2**)....5

Figure S3. ^{1}H-^{1}H COSY NMR spectrum (400 MHz, $CDCl_3$) of asperisocoumarin J (**2**)....6

Figure S4. HSQC NMR spectrum (400 MHz, $CDCl_3$) of asperisocoumarin J (**2**)....7

Figure S5. HMBC NMR spectrum (400 MHz, $CDCl_3$) of asperisocoumarin J (**2**)....8

Figure S6. ^{1}H NMR spectrum (500 MHz, $CDCl_3$) of asperisocoumarin K (**3**)....9

Figure S7 ^{13}C NMR spectrum (125 MHz, $CDCl_3$) of asperisocoumarin K (**3**)....10

Figure S8 DEPT-135 spectrum (125 MHz, $CDCl_3$) of asperisocoumarin K (**3**)....11

Figure S9. ^{1}H-^{1}H COSY NMR spectrum (500 MHz, $CDCl_3$) of asperisocoumarin K (**3**)....12

Figure S10. HSQC NMR spectrum (500 MHz, $CDCl_3$) of asperisocoumarin K (**3**)....13

Figure S11. HMBC NMR spectrum (500 MHz, $CDCl_3$) of asperisocoumarin K (**3**)....14

Figure S12. HR (+) ESI mass spectrum of asperisocoumarin J (**2**)....15

Figure S13. HR (+) ESI mass spectrum of asperisocoumarin K (**3**)....16

Figure S14. The necrosis of sowthistle leaf discs and wheat leaf segments under action asperisocoumarins J (**2**) (a) and K (**3**)(b)....17

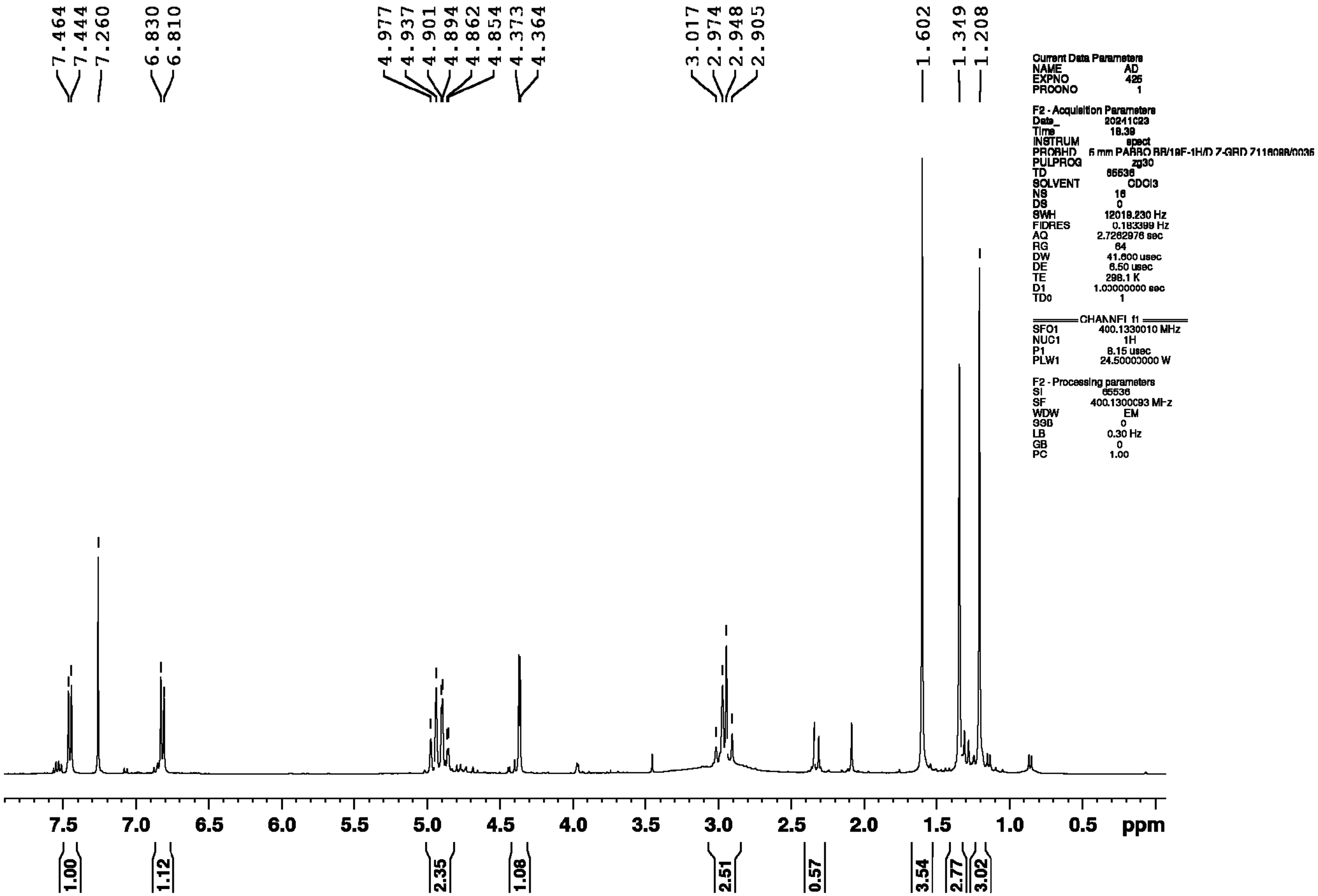

Figure S1. ^{1}H NMR spectrum (400 MHz, $CDCl_3$) of asperisocoumarin J (**2**)

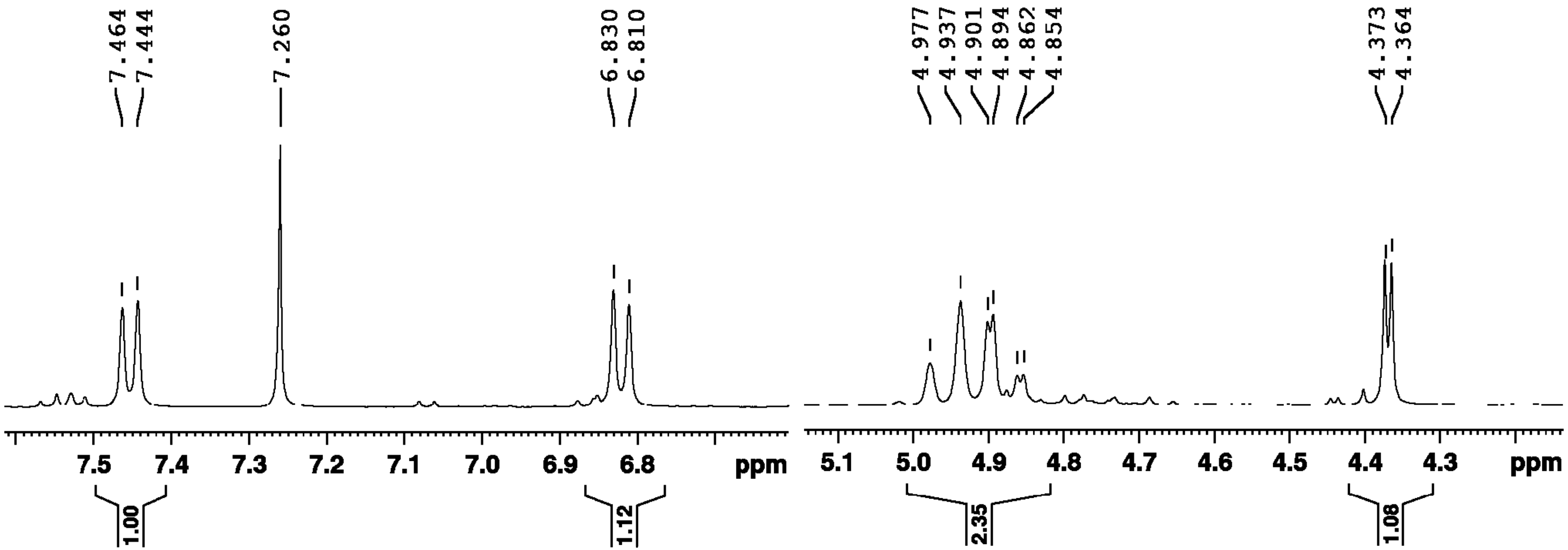

Continued Figure S1. ^{1}H NMR spectrum (400 MHz, $CDCl_3$) of asperisocoumarin J (**2**)

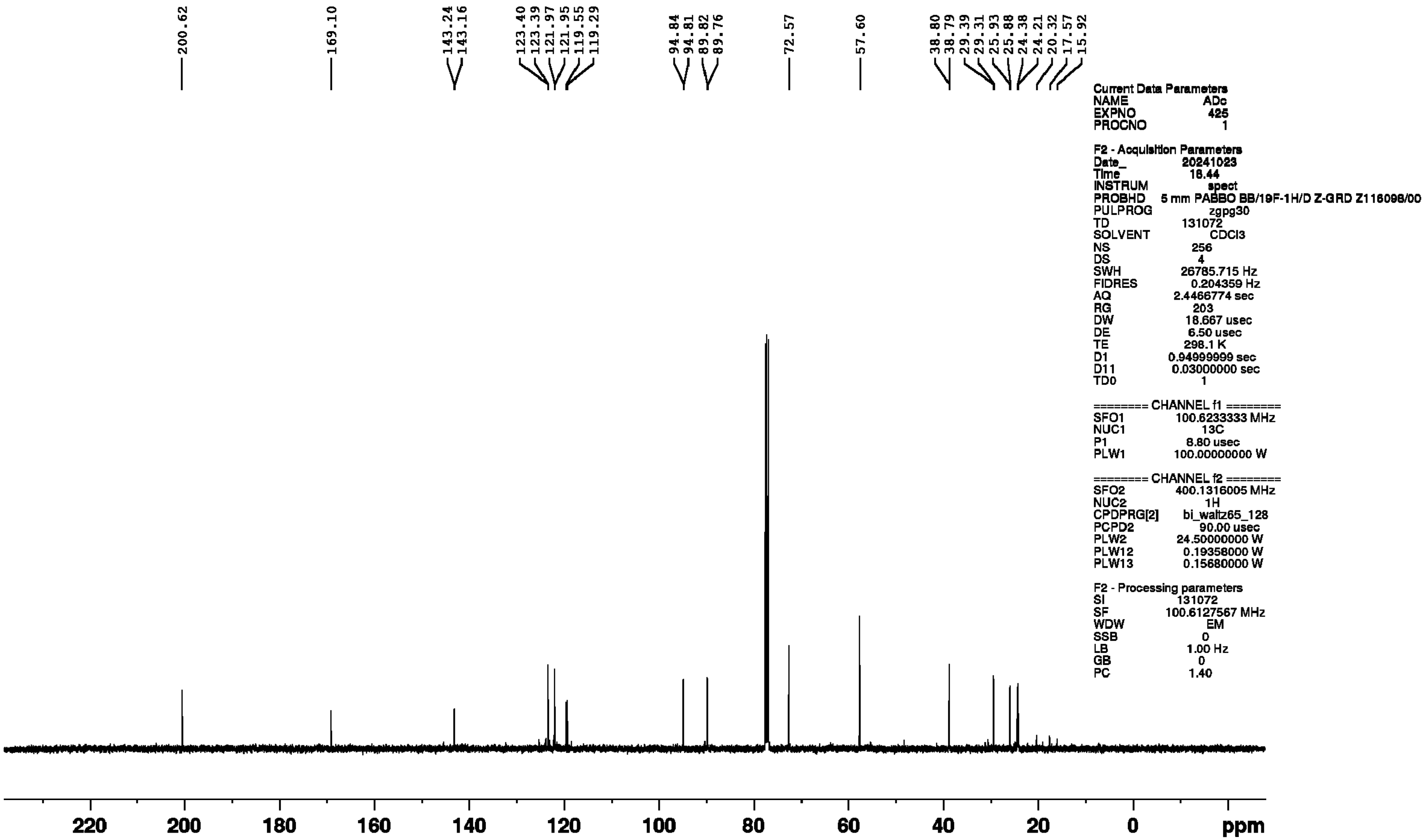

Figure S2. ^{13}C NMR spectrum (100 MHz, $CDCl_3$) of asperisocoumarin J (**2**)

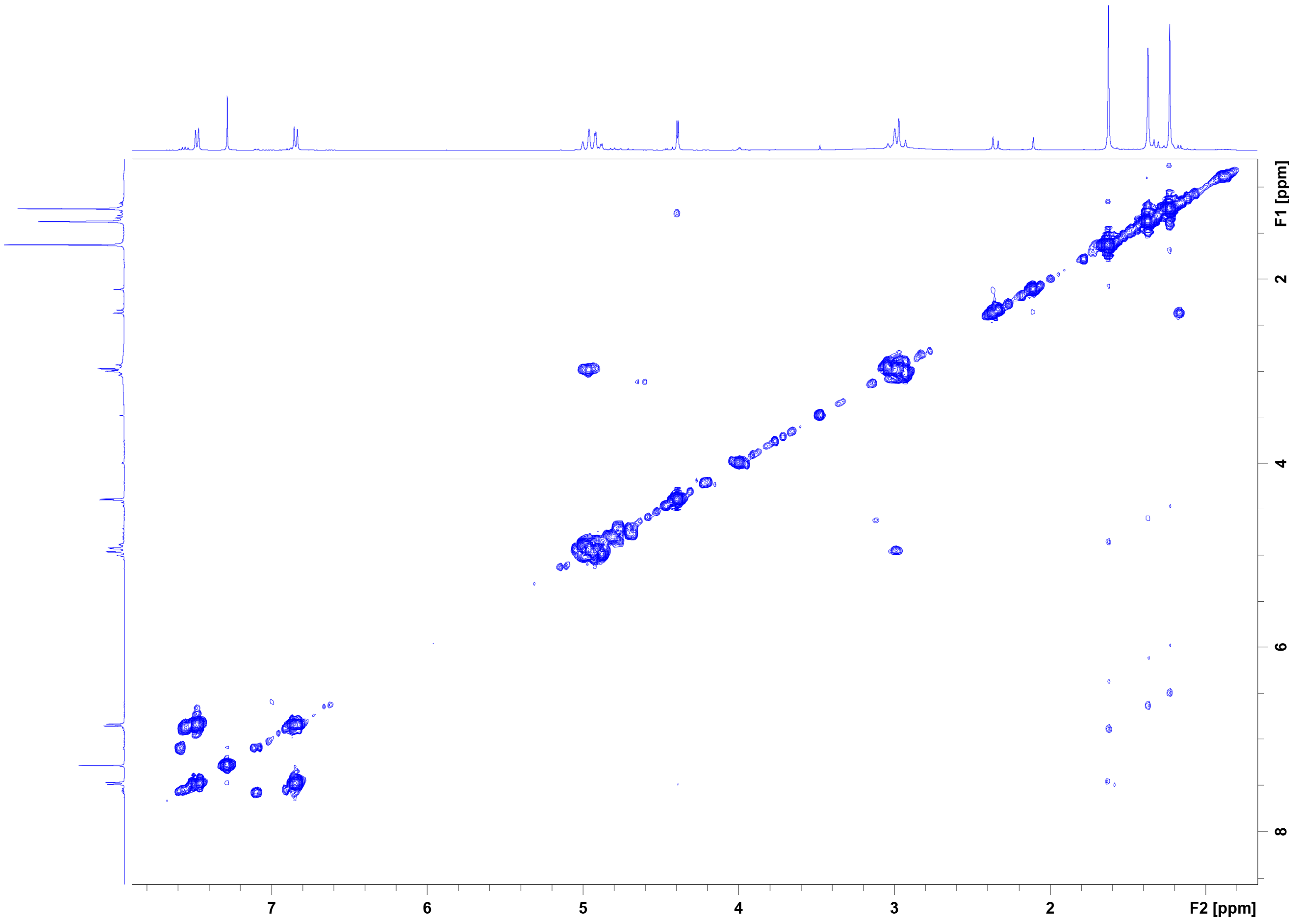

Figure S3. ^{1}H-^{1}H COSY NMR spectrum (400 MHz, $CDCl_3$) of asperisocoumarin J (**2**)

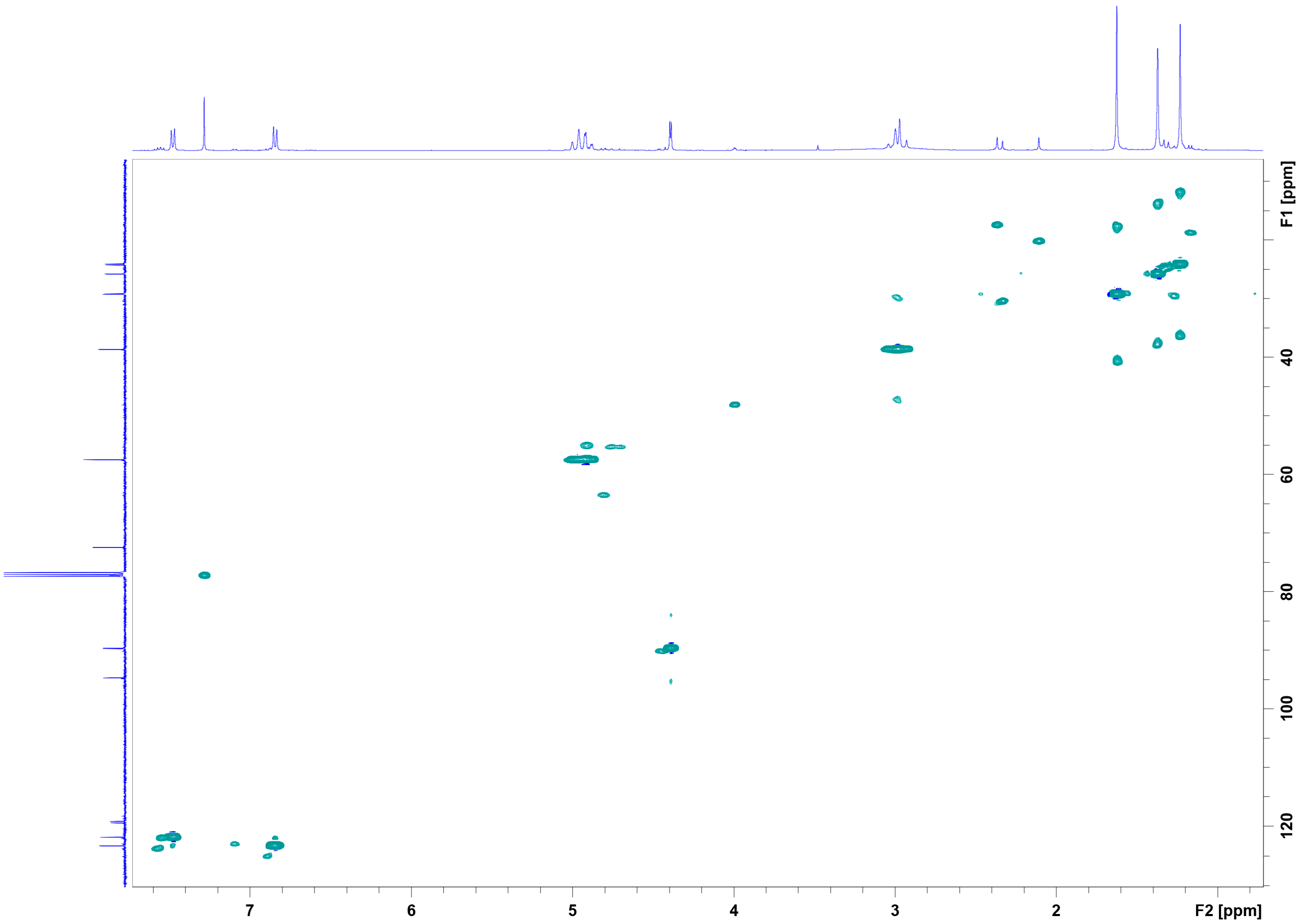

Figure S4. HSQC NMR spectrum (400 MHz, $CDCl_3$) of asperisocoumarin J (**2**)

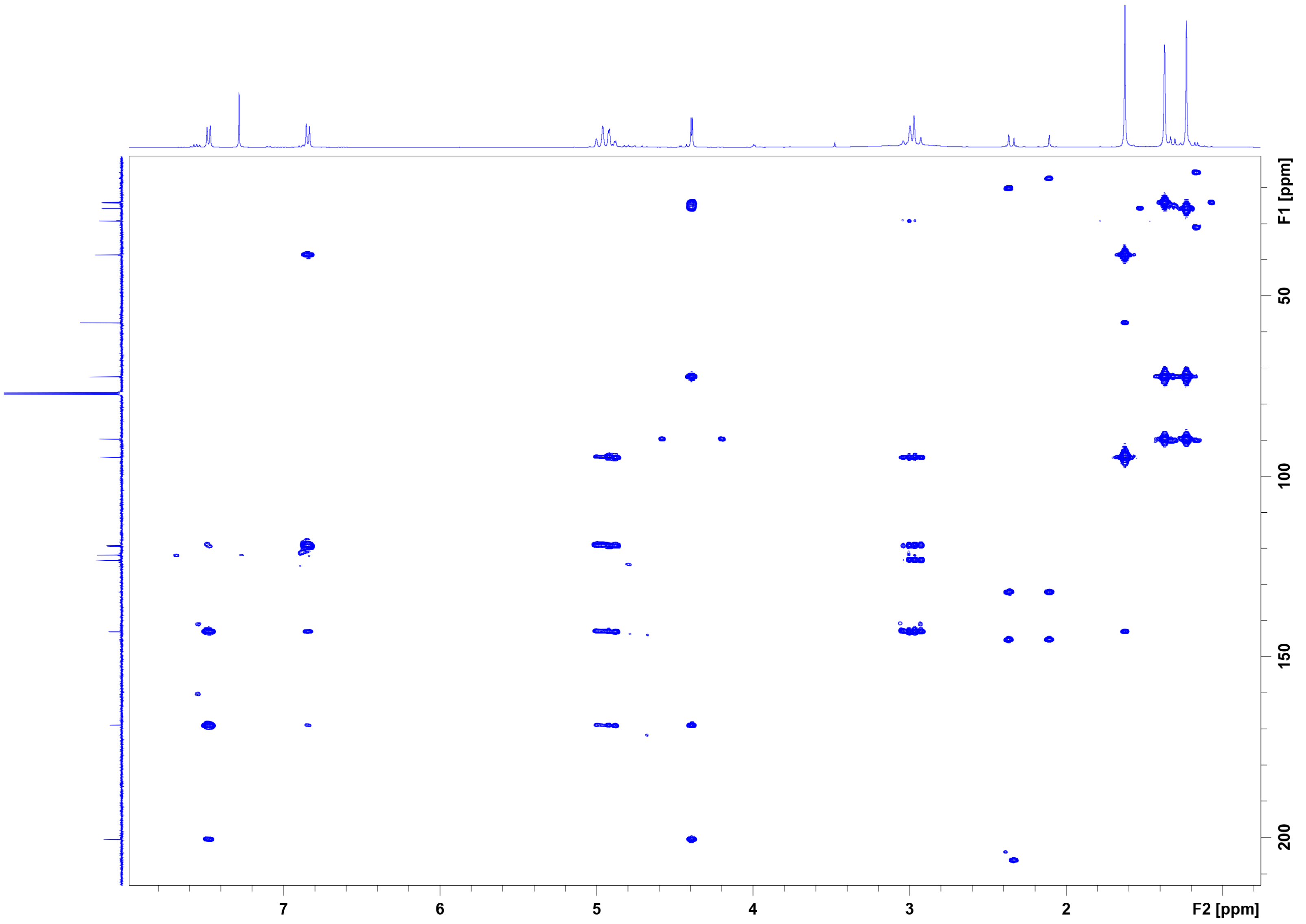

Figure S5. HMBC NMR spectrum (400 MHz, $CDCl_3$) of asperisocoumarin J (**2**)

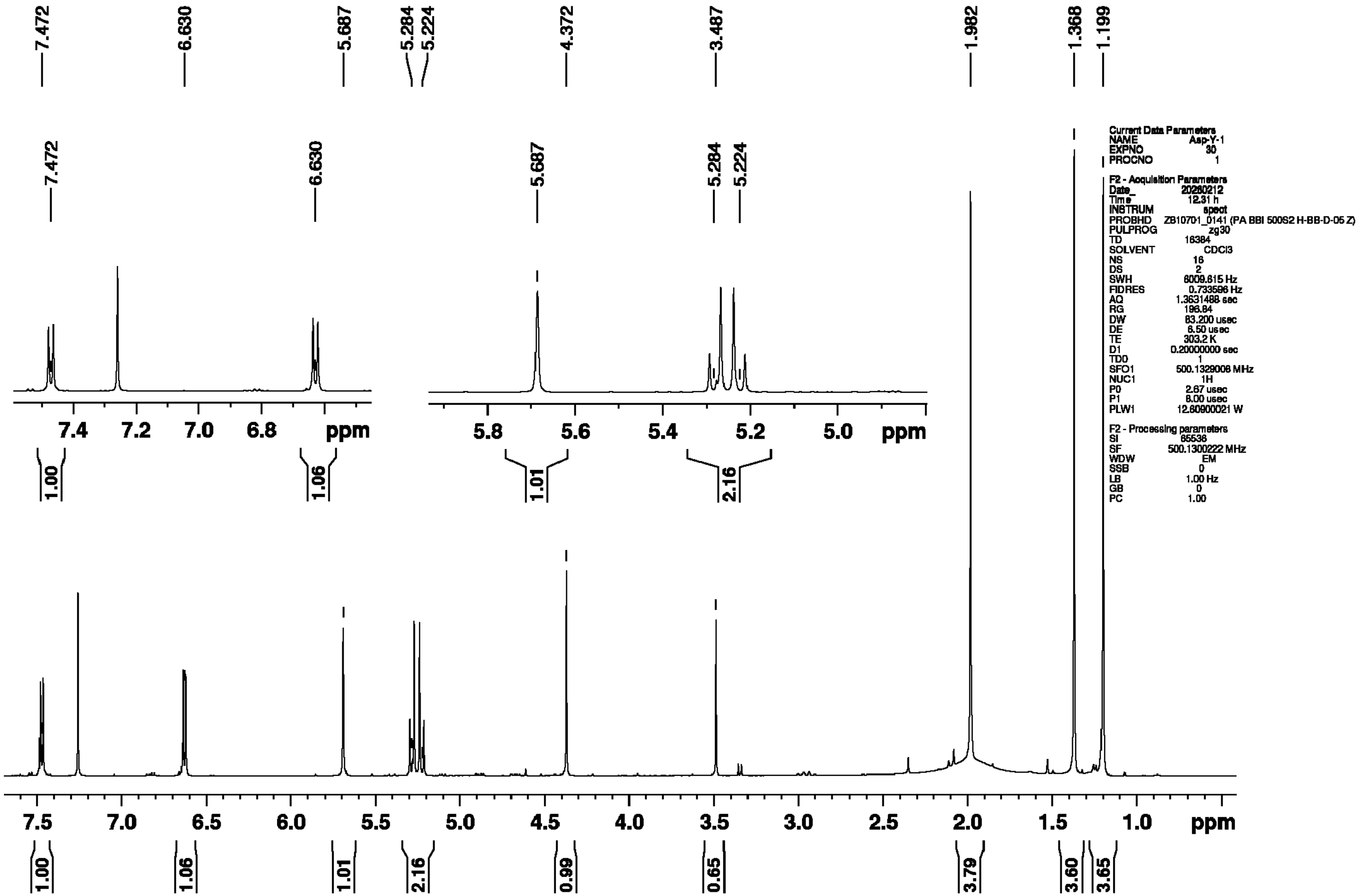

Figure S6. ^{1}H NMR spectrum (500 MHz, $CDCl_3$) of asperisocoumarin K (**3**)

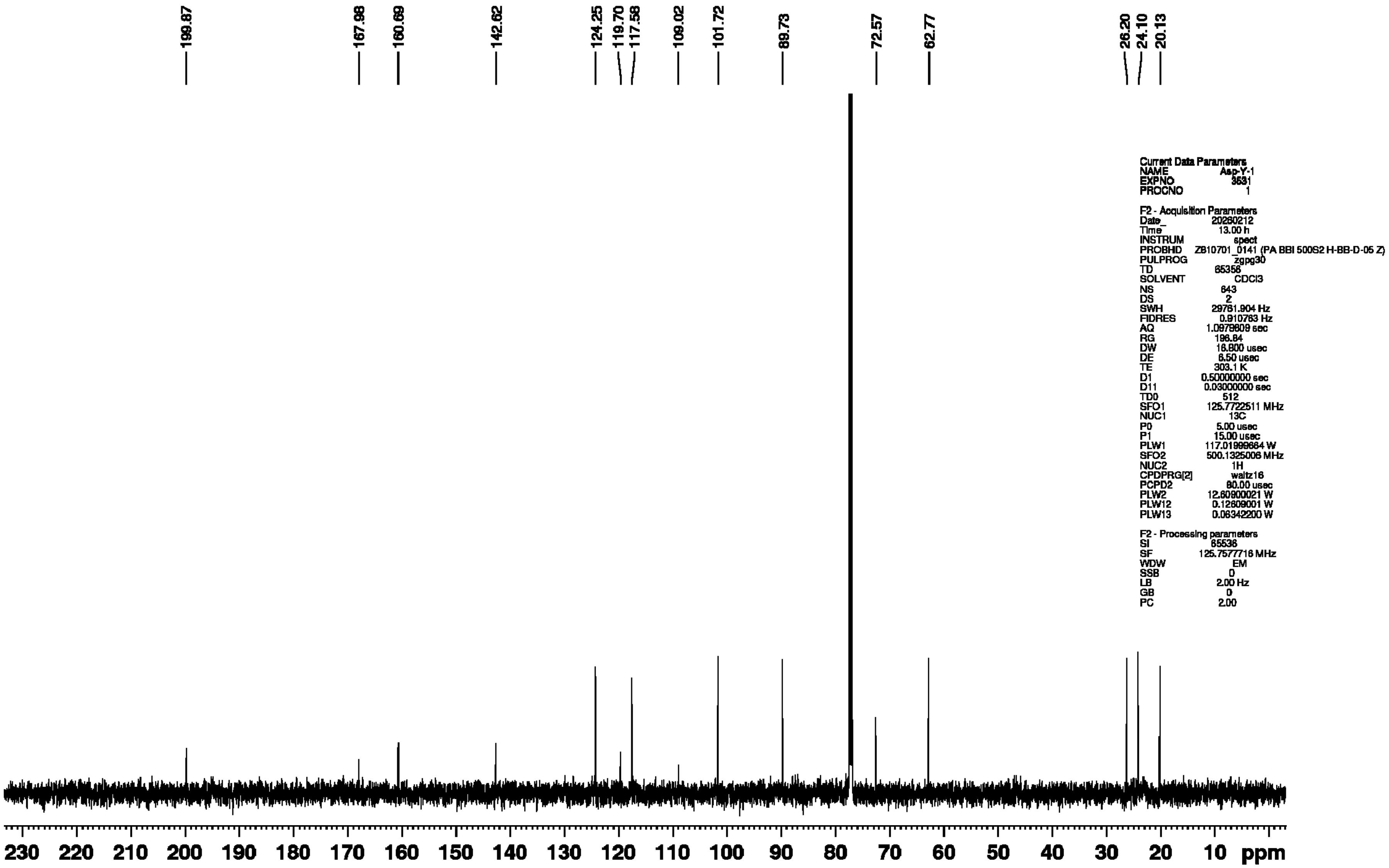

Figure S7 ^{13}C NMR spectrum (125 MHz, $CDCl_3$) of asperisocoumarin K (**3**)

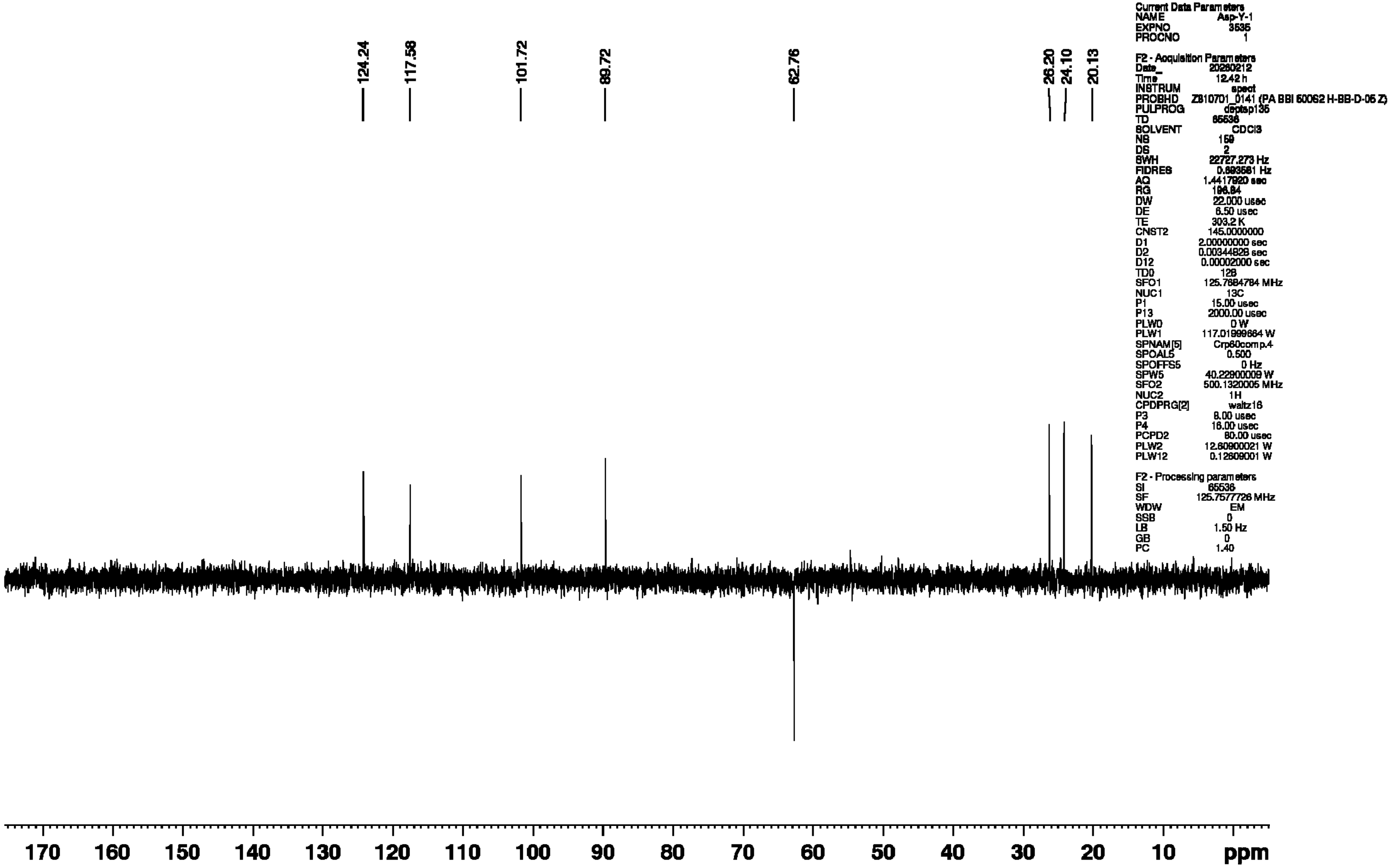

Figure S8 DEPT-135 spectrum (125 MHz, $CDCl_3$) of asperisocoumarin K (**3**)

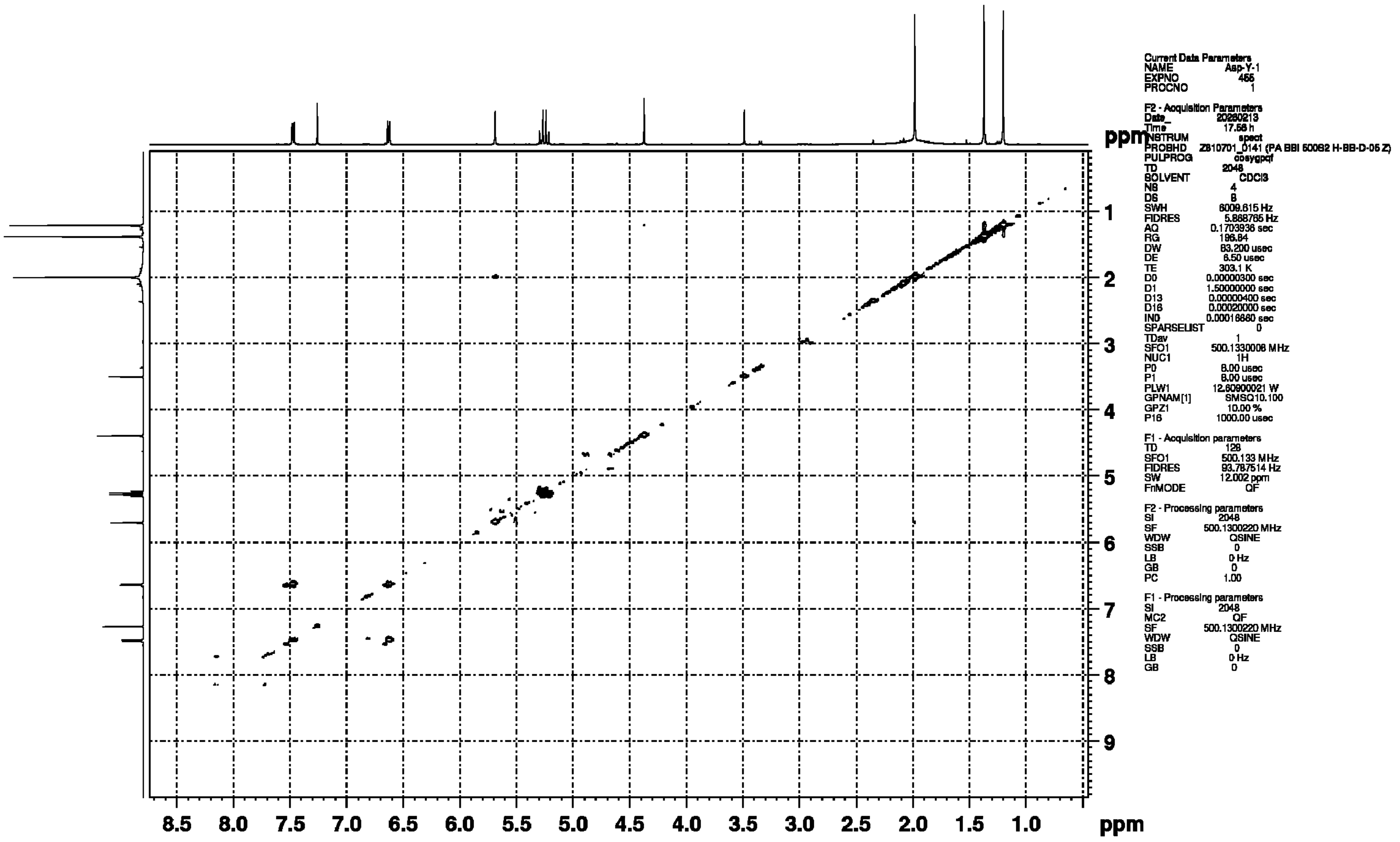

Figure S9. ^{1}H-^{1}H COSY NMR spectrum (500 MHz, $CDCl_3$) of asperisocoumarin K (**3**)

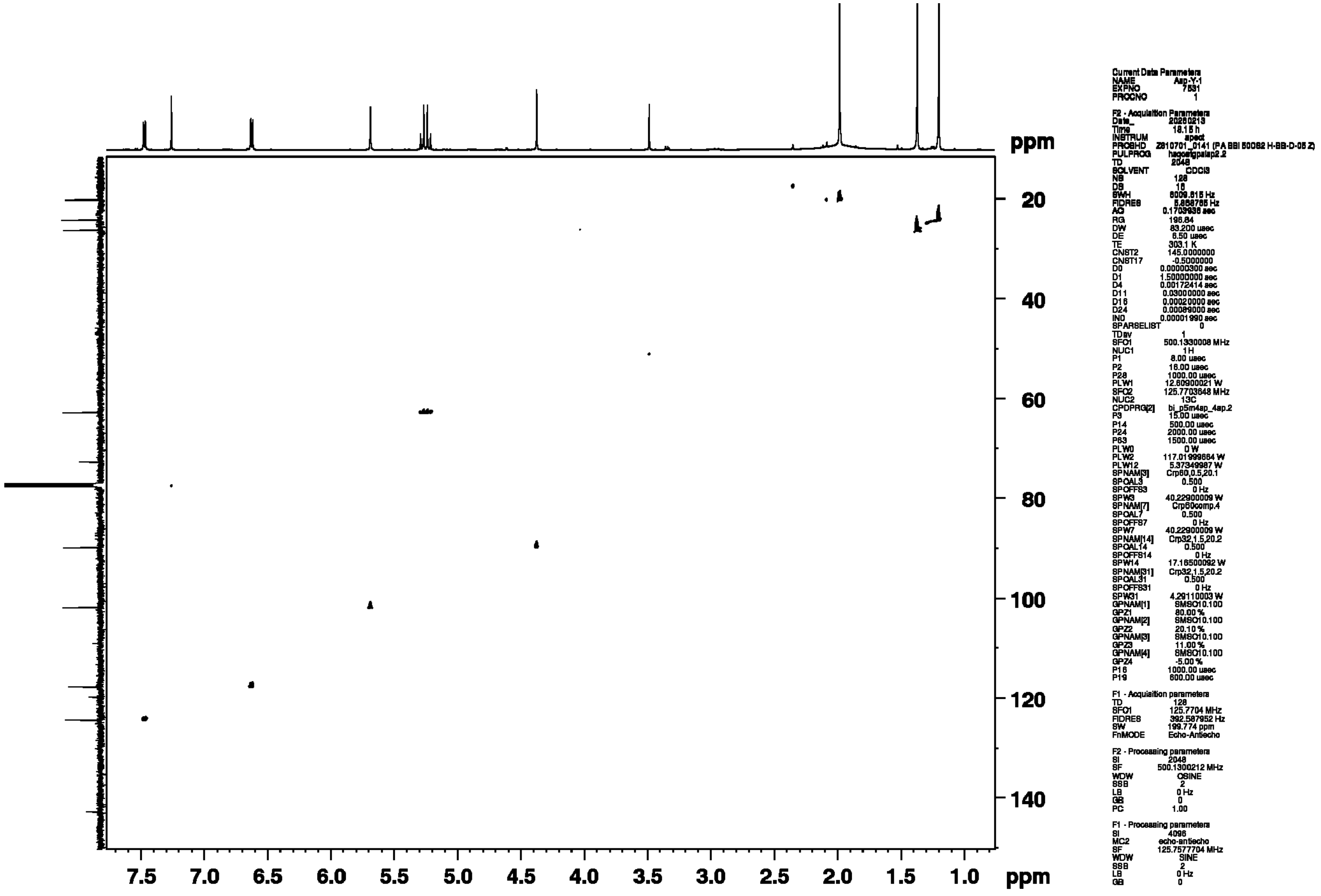

Figure S10. HSQC NMR spectrum (500 MHz, $CDCl_3$) of asperisocoumarin K (**3**)

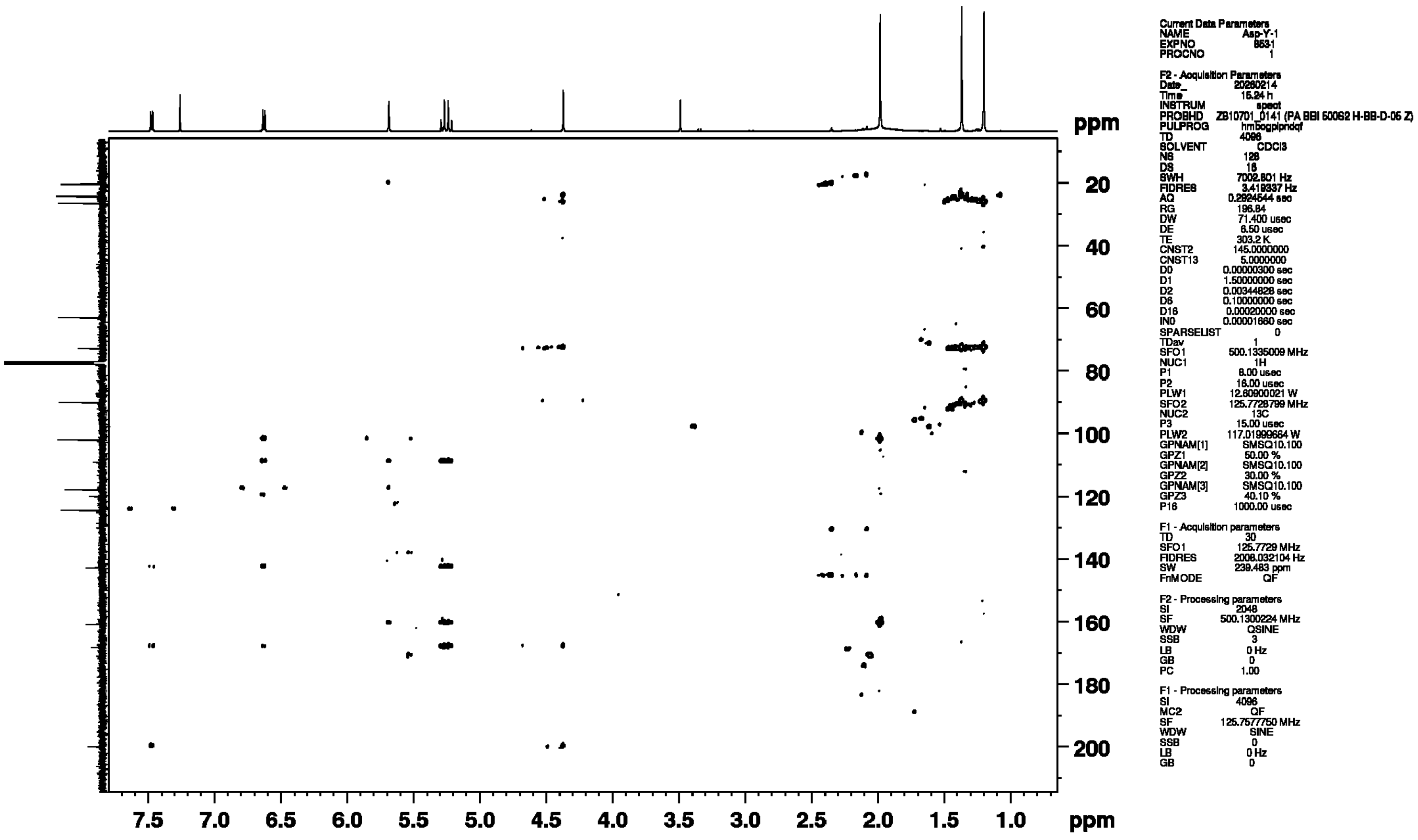

Figure S11. HMBC NMR spectrum (500 MHz, $CDCl_3$) of asperisocoumarin K (**3**)

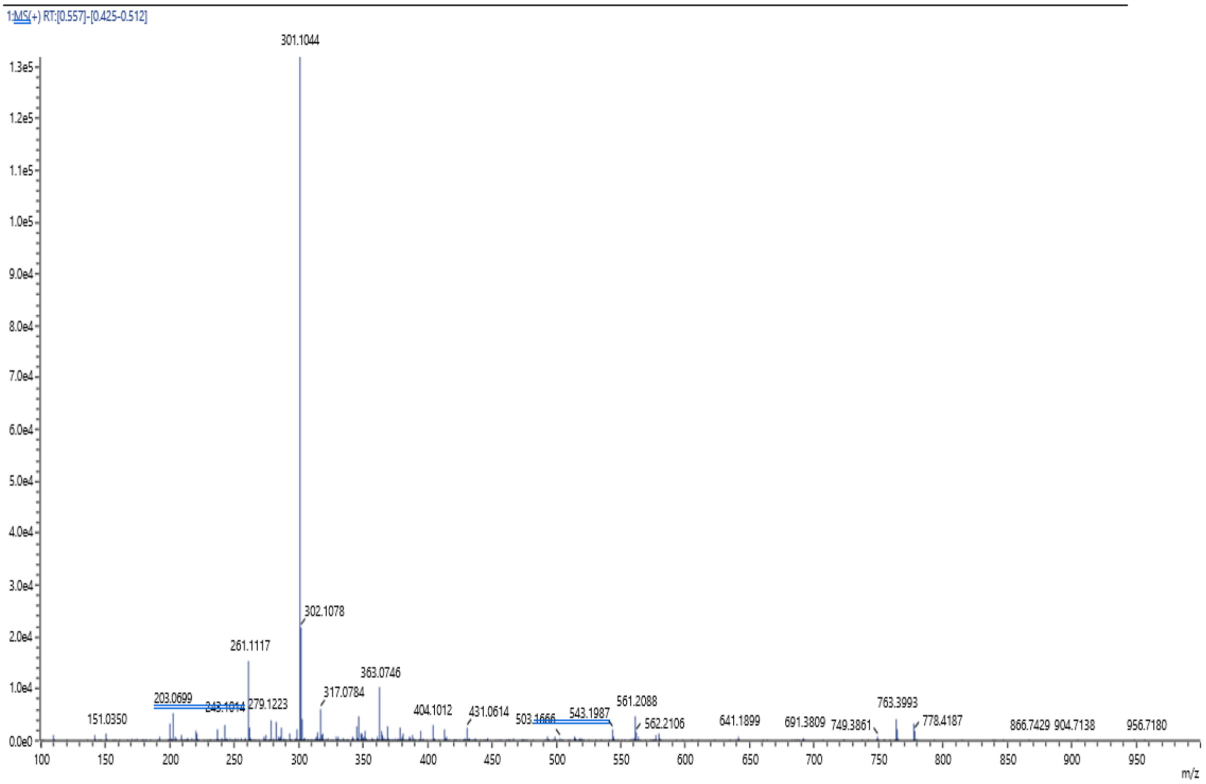

Figure S12. HR (+) ESI mass spectrum of asperisocoumarin J (**2**)

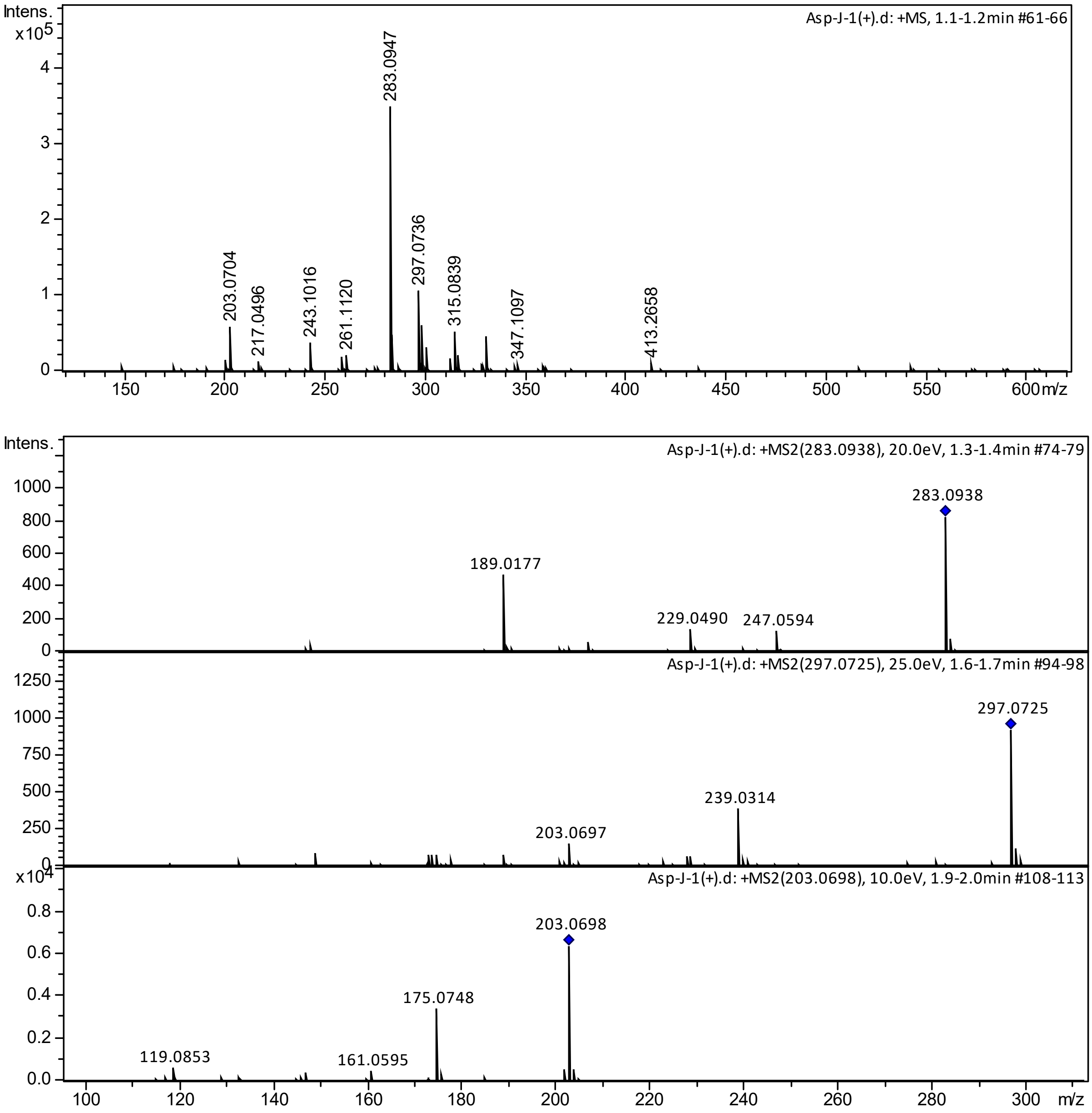

Figure S13. HR (+) ESI mass spectrum of asperisocoumarin K (**3**)

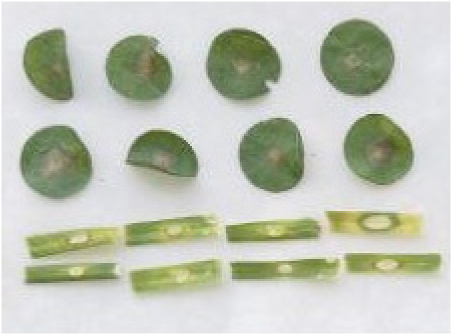

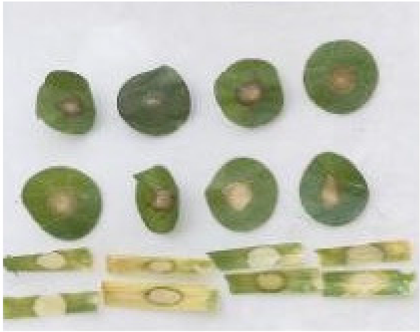

a) **b)**

Figure S14. The necrosis of sowthistle leaf discs and wheat leaf segments under action asperisocoumarins J (**2**) (a) and K (**3**) (b)
